# The N-linked glycosylation modifications in the hepatitis B surface protein impact cellular autophagy, HBV replication, and HBV secretion

**DOI:** 10.1371/journal.pone.0299403

**Published:** 2024-03-15

**Authors:** Patcharin Tepjanta, Kazuhito Fujiyama, Ryo Misaki, Ingorn Kimkong

**Affiliations:** 1 Department of Microbiology, Faculty of Science, Kasetsart University, Bangkok, Thailand; 2 International Center for Biotechnology (ICBiotech), Osaka University, Osaka, Japan; 3 Center for Advanced Studies in Tropical Natural Resources, National Research University – Kasetsart University, Bangkok, Thailand; Children’s National Hospital, George Washington University, UNITED STATES

## Abstract

N-linked glycosylation is a pivotal post-translational modification that significantly influences various aspects of protein biology. Autophagy, a critical cellular process, is instrumental in cell survival and maintenance. The hepatitis B virus (HBV) has evolved mechanisms to manipulate this process to ensure its survival within host cells. Significantly, post-translational N-linked glycosylation in the large surface protein of HBV (LHBs) influences virion assembly, infectivity, and immune evasion. This study investigated the role of N-linked glycosylation of LHBs in autophagy, and its subsequent effects on HBV replication and secretion. LHBs plasmids were constructed by incorporating single-, double-, and triple-mutated N-linked glycosylation sites through amino acid substitutions at N4, N112, and N309. In comparison to the wild-type LHBs, N-glycan mutants, including N309Q, N4-309Q, N112-309Q, and N4-112-309Q, induced autophagy gene expression and led to autophagosome accumulation in hepatoma cells. Acridine orange staining of cells expressing LHBs mutations revealed impaired lysosomal acidification, suggesting potential blockage of autophagic flux at later stages. Furthermore, N-glycan mutants increased the mRNA expression of HBV surface antigen (HBsAg). Notably, N309Q significantly elevated HBx oncogene level. The LHBs mutants, particularly N309Q and N112-309Q, significantly enhanced HBV replication, whereas N309Q, N4-309Q, and N4-112-309Q markedly increased HBV progeny secretion. Remarkably, our findings demonstrated that autophagy is indispensable for the impact of N-linked glycosylation mutations in LHBs on HBV secretion, as evidenced by experiments with a 3-methyladenine (3-MA) inhibitor. Our study provides pioneering insights into the interplay between N-linked glycosylation mutations in LHBs, host autophagy, and the HBV life cycle. Additionally, we offer a new clue for further investigation into carcinogenesis of hepatocellular carcinoma (HCC). These findings underscore the potential of targeting either N-linked glycosylation modifications or the autophagic pathway for the development of innovative therapies against HBV and/or HCC.

## Introduction

Hepatitis B virus (HBV) infection poses a significant global health threat, being a major contributor to liver diseases such as cirrhosis and hepatocellular carcinoma (HCC). According to the World Health Organization (WHO), an estimated 296 million individuals worldwide suffered from chronic hepatitis B (CHB) infection in 2019, leading to approximately 820,000 deaths [1, 2].

The HBV is an enveloped virus that comprises partially double-stranded circular DNA approximately 3200 base pairs in length. This DNA strand encodes four overlapping open reading frames (ORFs: S, C, P, and X) [3]. At present, HBV is classified into 10 genotypes (A–J) and more than 40 subgenotypes [4]. HCC pathogenesis in chronic HBV-infected patients has been studied extensively, and several important viral risk factors have been identified, such as high hepatitis B surface antigen (HBsAg) level, seropositivity of hepatitis B e antigen (HBeAg), high viral load, HBV intergenotypic recombination, and specific viral sequence mutations [5]. Previous studies on HBV recombination have identified different types of intergenotypic recombinants of genotypes B and C in CHB patients [6]. Furthermore, the presence of deletion/insertion mutations in the core promoter (CP) affects HBV transcriptional activity and HBx integrity, which are related to variations in disease progression [7]. In addition, several genetic characteristics, including mutations in the *preS1*, *preS2*, and *S* genes, have been shown to be associated with viral replication and progression of liver disease [8–10]. Interestingly, P overlaps with all other viral coding regions, and mutations in the polymerase gene may also affect the overlapping S gene, with implications for viral infectivity and the pathogenesis of liver disease. A recent study demonstrated that nucleotide mutations in the reverse transcriptase (RT) domain can encode premature stop codons in the corresponding S gene, resulting in truncation of the last dozen amino acids at the C-terminus of S proteins [11]. Additionally, cumulative evidence suggests that HBV genotypes play an essential role in promoting RT variation, which may alter their activity and drive the development of antiviral drug resistance [12].

HBV surface protein gene, a major viral protein secreted into patient serum, is characterized by three in-frame start codons that segment the gene into preS1, preS2, and S regions. These regions encode distinct proteins: the large surface (LHBs) protein comprises preS1, preS2, and S regions, the medium surface (MHBs) protein consists of preS2 and S regions, and the small surface (SHBs) protein is composed of the S region [13]. Post-translational modification (PTM) of the HBV large surface protein is integral to the regulation of cell membrane surface protein topologies and protein secretion [14]. Among these modifications, N-linked glycosylation plays a pivotal role. Co-translational N-linked glycosylation of the LHBs, MHBs, and SHBs occurs in the endoplasmic reticulum (ER) lumen, facilitating essential processes such as protein folding, stability, sorting, degradation, secretion, and modulation of the immune response [15]. The oligosaccharyltransferase complex catalyzes these reactions, transferring N-linked glycans to the Asn-X-Thr/Ser sequence, where X can be any amino acid except proline [16]. The LHBs protein, crucial for viral entry into the host cell, manifests as both a 42-kDa glycosylated (gp42) and a 39-kDa non-glycosylated (p39) form. Notably, N-linked glycans are located at N4, N112, and N309 of the preS1, preS2, and S regions, respectively, in the LHBs protein [15]. Previous studies emphasize the significance of N-linked glycosylation in HBV surface proteins for virion formation and secretion [17, 18]. Specifically, mutation at N112 in the preS region facilitates virion assembly and secretion, while mutation at N146 in the S region is essential for virion secretion [19]. Moreover, these modifications not only impact virion assembly and infectivity but also contribute to immune escape [18–21]. Recent studies further reveal an association between the N-glycans on LHBs and ER stress-mediated cell cycle dysregulation, cell proliferation, and the initiation of carcinogenic processes [22, 23]. Collectively, these findings highlight the significant roles of N-linked glycosylation modifications of LHBs in regulating HBV carcinogenesis.

Autophagy, an evolutionarily conserved catabolic degradation process, serves to maintain cellular homeostasis by eliminating cytoplasmic materials, including aggregated proteins, damaged organelles, or pathogens [24]. Increasing evidence suggests the involvement of autophagy in HBV replication and HBV-related hepatocarcinogenesis. HBV influences autophagy through various pathways, such as the interaction of the hepatitis B x protein (HBx) with phosphatidylinositol-3-kinase (PI3K) C3 or the activation of death-associated protein kinase in a Beclin-1-related pathway. Direct up-regulation of Beclin-1 expression by HBV also enhances autophagy, indicating that HBx induces autophagy at the initiation stage of autophagic progression [25–27]. The effects of autophagy on HBV exhibit diverse outcomes. Sir et al. demonstrated that the suppression of autophagy using 3-methyladenine or a specific small interfering RNA (siRNA) targeting ATG7 led to reduced HBV DNA replication [25]. In vivo studies using HBV transgenic mice with liver-specific knockout of the ATG5 gene confirmed the role of autophagy in significantly reducing HBV DNA levels [28]. Furthermore, autophagy plays a pivotal role in HBV-related immune responses. Research indicates that autophagy negatively impacts interferon signaling pathways, favoring HBV replication. Knockdown of the autophagy gene ATG12 using short hairpin RNA (shRNA) in HepG2.2.15 cells resulted in decreased HBV levels and increased expression of interferon-alpha (IFN-α), interferon-beta (IFN-β), and interferon-inducible (IFI) genes. This suggests that ATG12 plays a crucial role in HBV replication by downregulating the IFN pathway [29].

The objective of this study was to investigate the impact of N-linked glycosylation mutations in LHBs on autophagy in human hepatoma cells. Additionally, we aimed to assess their effects on HBV gene expression, DNA replication, secretion, and apoptosis.

## Materials and methods

### Cloning LHBs gene

The amplification of the L-HBsAg gene, which encodes the large surface proteins of HBV fused with 6xHis-tag sequences, was achieved through Polymerase Chain Reaction (PCR) using newly designed specific primers in this study. [Forward 5’-CAGCTAGCATGGGGACGAATCTTTC-3’ and Reverse 5’-GTAAGCTTTAGTGGTGATGGTGATGATGA-3’]. The PCR product was ligated to the pGEM-T vector. The plasmid containing the inserted DNA was introduced into *Escherichia coli* DH5α through the heat shock method. A preliminary screening of recombinant colonies was conducted by selecting white colonies, and their insert presence was confirmed through colony PCR, as well as verification via digestion with NheI and HindIII restriction enzymes, followed by sequencing.

### Site-directed mutagenesis

As previously reported, N-linked glycosylation occurs at N4, N112, and N309 in the HBV large surface protein (LHBs). Notably, a single mutation at the N-linked glycosylation site (N112Q) of LHBs has been reported to impede HBV release [15]. In continuation of this line of investigation, the present study delved deeper into the impact of N-linked glycosylation modifications of LHBs. Through a PCR-based site-directed mutagenesis method, we introduced single, double, and triple glycosylated mutants of LHBs by substituting asparagine residues at amino acid positions 4, 112, and 309 with glutamine. The mutagenic primers utilized for this purpose are detailed in Table 1. Mutants were generated using the QuikChange II site-directed mutagenesis kit following the manufacturer’s instructions. The amino acid mutations were validated through DNA sequencing. The resulting constructs comprised N4Q, N112Q, N309Q, N4-112Q, N4-309Q, N112-309Q, and N4-112-309Q, respectively.

**Table 1:** Mutagenic primers.

Primer name	Sequences (5’- 3’)
N4Q	F: GGGACGCAGCTTTCTGTTCCCAACCCT R: AGAAAGCTGCGTCCCCATGCTAGCTG
N112Q	F: CAGTGGCAGTCCACTGCCTTCCACCAA R: AGTGGACTGCCACTGCACGGCCTGAGG
N309Q	F: GACGGACAGTGCACCTGTATTCCCATC R: GGTGCACTGTCCGTCCGAAAGGTTTGGT

### Subcloning the constructs into a mammalian expression vector

The pGEM-T vector carrying either the wild-type or mutant forms of LHBs underwent digestion using NheI and HindIII restriction enzymes. The resulting purified DNA fragment (LHBs) was subsequently ligated into the pcDNA3.1 mammalian expression vector and transformed into *Escherichia coli* DH5α competent cells. Transformants were selected on media containing ampicillin, and their inserts were rigorously examined through colony PCR, restriction digestion, and sequencing to ensure the proper orientation of the cloned gene.

### Cell culture

The human embryonic kidney 293T cell line (HEK293T, ATCC) was cultured in Dulbecco’s Modified Eagle’s Medium (DMEM; Gibco, USA) supplemented with 10% heat-inactivated fetal bovine serum (FBS; Gibco, USA) and 1% penicillin-streptomycin (10,000 U/ml, Gibco, USA). Human hepatoma cell lines, Huh-7, HepG2, and the HBV-producing HepG2.2.15 from Professor Nattiya Hirankarn, Faculty of Medicine, Chulalongkorn University, were cultured in RPMI 1640 medium (Gibco, USA) supplemented with 10% FBS (Gibco, USA) and 1% penicillin-streptomycin (10,000 U/ml, Gibco, USA). For the maintenance of HepG2.2.15 cells, G418 (Merck, USA) was added to the medium at a final concentration of 380 mg/L. All cultures were incubated at 37°C in a humidified atmosphere with 5% CO_2_.

### Transient transfection

The cells were seeded in six-well plates and allowed to grow until reaching 60–70% confluence. DNA transfection was carried out using Lipofectamine^™^ 3000 transfection reagent (Invitrogen, USA) following the manufacturer’s guidelines. As a control, an empty vector pcDNA3.1 was employed. The cells were then maintained in a 37°C incubator with a 5% CO_2_ atmosphere for 72 hours.

### Autophagy induction by cell starvation

The cells underwent three washes with pre-warmed PBS and were subsequently incubated in Earle’s balanced salts solution (EBSS; composed of 117 mM NaCl, 1.8 mM CaCl_2_, 0.8 mM MgSO_4_, 5.3 mM KCl, 5.6 mM glucose, 1 mM NaH_2_PO_4_-H_2_O, and 26 mM NaHCO_3_, pH 7.4) at 37°C for 6 hours.

### Protein extraction

At the 72-hour post-transfection time point, cells were washed once with PBS before the addition of 300 μl of CytoBuster^™^ protein extraction reagent, a commercial lysis buffer (Merck, Germany). The mixture was allowed to extract at room temperature for 5 minutes. Following extraction, cells were scraped using a cell scraper and pooled in the solution. The samples were then transferred to an appropriate tube and centrifuged for 5 minutes at 16,000 x g at 4°C. The resulting lysates containing proteins were transferred to new tubes, and protein concentration was determined using a Pierce^™^ BCA protein assay kit (Thermo Fisher Scientific, USA) following the manufacturer’s protocols.

### Detection of LHBs expression and N-linked glycosylation

HEK293T cells were used to investigate the expression profile of LHBs and verify N-linked glycosylation. To remove N-linked glycans from LHBs with N-linked glycosylation sites, Peptide-N-glycosidase F (PNGase F) was used following the manufacturer’s instructions. In brief, cell lysates were boiled for 10 minutes in glycoprotein denaturing buffer (0.5% SDS, 40 mM DTT). Subsequently, GlycoBuffer 2, 10% NP-40, and PNGase F were added, and the reaction mixture was incubated at 37°C for 1 hour. The treated and untreated samples were then separated on 12% SDS-polyacrylamide gels and transferred to polyvinylidene difluoride (PVDF) membranes. The expression of LHBs, both untreated and treated with PNGase F, was assessed by western blotting using a mouse monoclonal anti-Hep B preS1 Antibody (AP2) (1:1000; sc-57762; Santa Cruz Biotechnology, USA) as the primary antibody. Detection was achieved using mouse IgGκ binding protein (m-IgGκ BP) conjugated to horseradish peroxidase (HRP) (1:5000; sc-516102; Santa Cruz Biotechnology, USA) in combination with SuperSignal West Femto chemiluminescent substrate (Thermo Scientific, USA). The membranes were visualized using a ChemiDoc imaging system (Bio-Rad, USA).

### RNA extraction and cDNA synthesis

At the 72-hour post-transfection time point, total RNA was extracted using cold Trizol (Invitrogen, USA) and absolute ethanol (Merck, Germany). Subsequently, RNA was isolated and purified using the Direct-zol RNA Miniprep Kit (Zymo Research, USA). The quality and quantity of RNA were assessed using a nanodrop spectrophotometer (NanoPhotometer N60/N50, Implen GmbH, Germany) at 260/280 nm. First-strand cDNA was synthesized from 1 μg of total RNA using the iScript cDNA Synthesis Kit (Bio-Rad, USA) following the manufacturer’s instructions. The resulting cDNA was stored at -20°C until needed for use in RT-qPCR.

### Analysis of relative gene expression levels of autophagy and apoptosis, by Reverse transcription-quantitative PCR (RT-qPCR)

The RT-qPCR was conducted using a CFX96 real-time PCR detection system (Bio-Rad, USA) with iTaq Universal SYBR Green Supermix (Bio-Rad, USA). The reaction mixture consisted of 2 μl of cDNA template, 10 μL of iTaq Universal SYBR Green Supermix (2X) (Bio-Rad, USA), and 0.3 μM of each forward and reverse primer (Table 2), adjusted to a final volume of 20 μl with deionized water. The thermal cycling conditions included pre-incubation at 95°C for 30 seconds, followed by 40 cycles of amplification at 95°C for 5 seconds and 60°C for 30 seconds. The final melting step involved a gradient from 65°C to 95°C with a ramping rate of 0.5°C/5 seconds. Relative gene expression levels for autophagy and apoptosis were calculated using the 2^-ΔΔCt^ method, where ΔCt represents the differences in the cycle threshold number between the target gene and *β-actin*, and ΔΔCt represents the relative change in differences between the wild-type (WT) and mutant groups.

**Table 2:** List of primers used for RT-qPCR.

Gene	Primer Sequences (5’- 3’)
**Autophagy-related genes**	
*mTOR*	F: GCAGATTTGCCAACTATCTTCGG R: CAGCGGTAAAAGTGTCCCCTG
*AMPK*	F: GTCATGATAGCTTGCATAAATGGTG R: AGTTGAATAGAACAAGCCCTGGAC
*BECN1*	F: CCATGCAGGTGAGCTTCGT R: GAATCTGCGAGAGACACCATC
*LC3-II*	F: GATGTCCGACTTATTCGAGAGC R: TTGAGCTGTAAGCGCCTTCTA
*ATG9A*	F: TCATGCAGTTCCTCTTTGTGG R: TCTGGCAGAGTGACCTTG
*ATG12*	F: TAGAGCGAACACGAACCATCC R: CACTGCCAAAACACTCATAGAGA
*ATG16L1*	F: AACGCTGTGCAGTTCAGTCC R: AGCTGCTAAGAGGTAAGATCCA
**Apoptosis-related genes**	
*BAX*	F: ATGTTTTCTGACGGCAACTTC R: AGTCCAATGTCCAGCCCAT
*Caspase-3*	F: TGTTTGTGTGCTTCTGAGCC R: ACGGCAGGCCTGAATAATGA
*AIFM1*	F: GTTCCAGCGATGGCATGTTC R: TAGGCACCAGCTCCTACTGT
*BCL2*	F: ATGTGTGTGGAGAGCGTCAA R: GCCGTACAGTTCCACAAAGG
**Housekeeping gene**	
*β-actin*	F: GTGGTGGTGAAGCTGTAGCC R: ACCAACTGGGACGACATGGAGAA

### Western blotting

Equal protein amounts were loaded onto 12% SDS-polyacrylamide gels and transferred to polyvinylidene difluoride (PVDF) membranes (Thermo Scientific, USA) via electroblotting at 100 V for 45 minutes. Afterward, the membranes were blocked for 1 hour in 5% non-fat milk in Tris-buffered saline containing 0.1% Tween-20 (TBS-T) at room temperature. Subsequently, they were incubated overnight at 4ºC with specific primary antibodies: rabbit polyclonal anti-LC3A/B (1:1000; 4108; Cell Signaling Technology, USA), mouse monoclonal anti-GAPDH (1:5000; sc-47724; Santa Cruz Biotechnology, USA), and Cocktail Apoptosis kit (ab136812 Abcam, USA). Following three washes with TBS-T, the membranes were incubated with the corresponding horseradish peroxidase-conjugated secondary antibodies: goat anti-rabbit (1:5000 7074; Cell Signaling Technology, USA) or goat anti-mouse (1:5000; sc-516102; Santa Cruz Technology, USA) for 1 hour at room temperature. Protein-specific bands were visualized using SuperSignal West Femto chemiluminescent substrate (Thermo Scientific, USA) and captured with a ChemiDoc imaging system (Bio-Rad, USA). Quantification was performed using ImageJ analysis software (Wayne Rasband, NIH, USA), with GAPDH detection serving as the control for total protein loading.

### Analysis of lysosomal acidification by Acridine orange staining (AO)

Huh-7 cells (3×10^4^) were cultured on 8-chamber culture slides and transfected with plasmids expressing either wild-type (WT) or N-glycan mutants of LHBs. After 72 hours of transfection, the cells were fixed with 4% formaldehyde for 10 minutes at room temperature, washed with PBS, and then incubated for 15 minutes at 37°C with 4 μg/ml acridine orange (AO) (Sigma-Aldrich, USA) in the dark. Following the removal of excess AO by washing with PBS, AO fluorescence was examined using an Olympus FV 3000 confocal microscope with a 488-nm (green) or 561-nm (red) laser.

### Detection of HBV replication and gene expression

At 72 hours post-transfection into HepG2.2.15 cells, HBV replicative intermediates (RIs) from intracellular core particles were extracted from transfected-HepG2.2.15 cells with or without the autophagy inhibitor 3-methyladenine (3-MA) treatment, as previously described [30], with slight modifications. Briefly, cells were lysed with lysis buffer (50 mM Tris-HCl, pH 7.4, 150 mM NaCl, 5 mM MgCl_2_, and 1% NP-40) for 10 minutes. The cytoplasmic fraction was separated from the nuclear fraction through centrifugation. Supernatants were then transferred to a new tube, mixed with 10 mM MgCl_2_ and 500 μg/ml DNase I (Sigma-Aldrich, USA), and incubated for 1 hour at 37°C. The reaction was stopped with 25 mM EDTA. Subsequently, sodium dodecyl sulfate and proteinase K (Vivantis, Malaysia) were added separately and incubated for 1.5 hours at 56°C. Viral DNA was then purified through phenol-chloroform extraction and isopropanol precipitation. HBV progeny DNA was extracted from virions in the cell culture supernatant using a QiAamp DNA blood mini kit (Qiagen, Germany). The detection of HBV DNA was performed using quantitative real-time PCR (qPCR), and the quantification of HBV DNA was determined by comparison to a standard curve. Additionally, HBV transcripts were extracted from HepG2.2.15 cells and detected using RT-qPCR. The mRNA levels were normalized to the beta-actin expression level. Primer sequences can be found in Table 3.

**Table 3:** Primer sequences for detecting HBV DNA and HBV RNA by real-time PCR.

Primer name	Primer Sequences (5’-3’)
HBV DNA	F: GTTGCCCGTTTGTCCTCTAATTC R: GGAGGGATACATAGAGGTTCCTTGA
HBx	F: CCGTCTGTGCCTTCTCATCT R: ATCTCCTCCCCCAACTCCTC
HBsAg	F: GTTGCCCGTTTGTCCTCTAATTC R: GGAGGGATACATAGAGGTTCCTTGA

### Statistical analysis

All experiments were independently conducted three times. Data were visualized using GraphPad Prism 9.0 software (GraphPad Software, USA) and subjected to statistical analysis using SPSS Statistics 25 software (IBM Corporation, United States). Results are presented as means ± standard errors of the means (SEM). Statistical significance was assessed through Student’s t-test or one-way analysis of variance (ANOVA) followed by Tukey’s multiple comparison test. A *p*-value less than 0.05 was considered statistically significant.

## Results

### Amino acid asparagine at site 309 of LHBs is a key site of N-linked glycosylation

**Fig 1:**
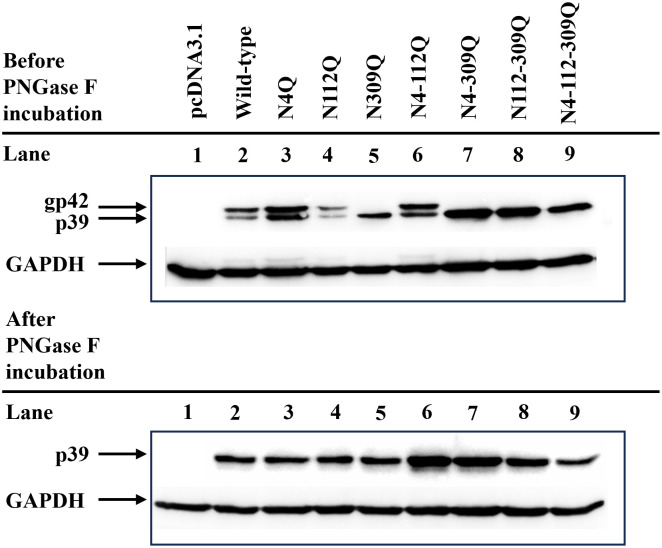
Detection of N-linked glycosylation in the mutated LHBs. HEK293T cells were transfected with plasmids expressing either WT LHBs or N-glycan mutants.

To assess the expression pattern of LHBs and confirm the presence of N-linked glycans, HEK293T cells were transfected with plasmids carrying either wild-type (WT) or N-glycan mutants of LHBs. Cell lysates were mock-treated or treated with PNGase F (Peptide: N-glycosidase F), an enzyme cleaving high-mannose, hybrid, and complex oligosaccharides from N-linked glycoproteins, followed by LHBs-specific immunoblotting. The WT LHBs protein and its N4Q, N112Q, and N4-112Q mutants exhibited two bands representing the glycosylated (gp42) and non-glycosylated (p39) forms. In contrast, the N309Q, N4-309Q, N112-309Q, and N4-112-309Q mutants displayed only a non-glycosylated band (gp39) (Fig 1), indicating the importance of asparagine residue 309 in the N-linked glycosylation of LHBs. After PNGase F treatment, both WT LHBs and their mutants showed a single non-glycosylated band (gp39) (Fig 1), confirming that the observed differences in molecular weight were attributed to N-linked glycan additions on the protein.

After 72 hours, the cell lysates were subjected to mock digestion or treated with PNGase F, followed by western blot analysis using an anti-Hep B preS1 antibody. The non-glycosylated p39 and single-glycosylated gp42 forms of the LHBs are denoted. Cells transfected with the pcDNA3.1 plasmid served as a negative control.

### N-linked glycosylation mutations of LHBs increase autophagic response

**Fig 2:**
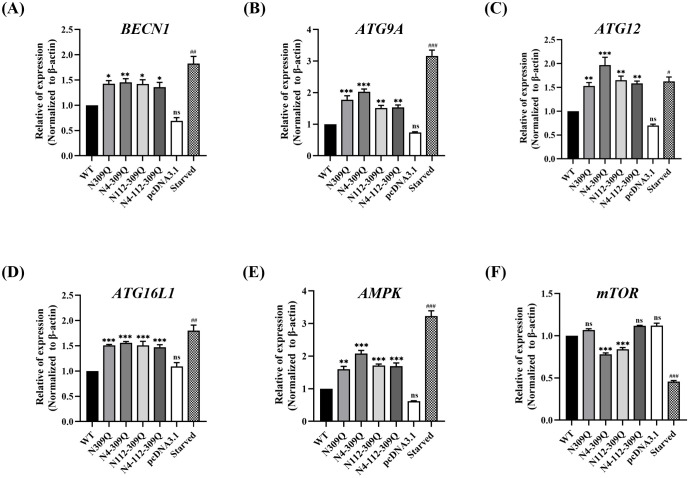
Relative mRNA levels of autophagic markers in HepG2 cells. The relative mRNA levels of (A) *BECN1*, (B) *ATG9A*, (C) *ATG12*, (D) *ATG16L1*, (E) *AMPK*, and (F) *mTOR* in HepG2 cells were determined 72 hours after transfection with LHBs mutants compared to WT. Nutrient starvation-induced autophagy in HepG2 cells, compared to that in non-starved cells, served as a positive control. *β-Actin* was used as an internal control. Data are plotted as the mean ± SEM. *p < .05; **p < .01; ***p < .001; ^#^*p* < .05; ^##^*p* < .01; ^###^*p* < .001; ns, not significant.

To elucidate the impact of N-linked glycosylation mutations in HBV large surface protein (LHBs) on autophagy in HCC cell line, HepG2 cells were transfected with plasmids expressing either N-glycosylated LHBs (WT) or N-glycan mutants of LHBs at N309Q, N4-309Q, N112-309Q, and N4-112-309Q. Analysis of mRNA expression levels of autophagic markers, including *BECN1* (an essential autophagy regulator), *ATG9A*, *ATG12*, *ATG16L1*, *AMPK*, and *mTOR*, at 72 hours post-transfection revealed significant upregulation in HepG2 cells transfected with all LHBs mutants. In contrast, the mRNA levels of mTOR were downregulated in double mutant-transfected cells compared to the WT (Fig 2).

**Fig 3:**
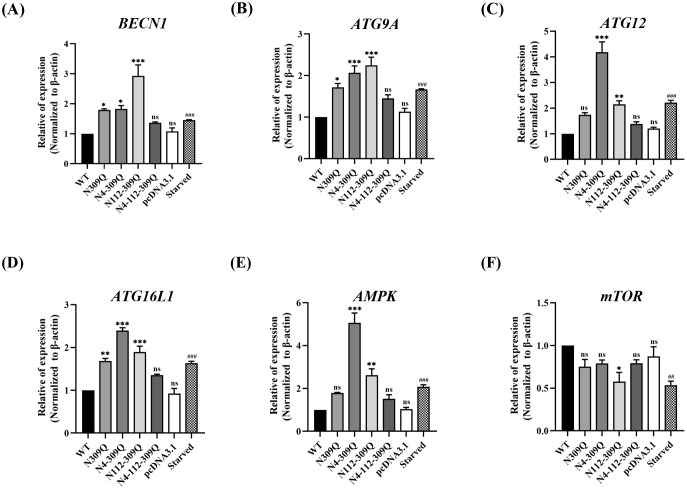
Relative mRNA levels of autophagic markers in Huh-7 cells. The relative mRNA levels of (A) *BECN1*, (B) *ATG9A*, (C) *ATG12*, (D) *ATG16L1*, (E) *AMPK*, and (F) *mTOR* in Huh-7 cells were determined 72 hours after transfection with LHBs mutants compared to WT. Nutrient starvation-induced autophagy in HepG2 cells, compared to that in non-starved cells, served as a positive control. *β-Actin* was used as an internal control. Data are plotted as the mean ± SEM. *p < .05; **p < .01; ***p < .001; ^#^*p* < .05; ^##^*p* < .01; ^###^*p* < .001; ns, not significant.

In a similar experiment conducted using Huh-7 cells, the results demonstrated significant increases in mRNA levels of *BECN1*, *ATG9A*, *ATG12*, *ATG16L1*, and *AMPK*, while *mTOR* mRNA levels were decreased by N112-309Q mutant-transfected cells compared to WT (Fig 3). The induction of autophagy by LHBs mutants in both HCC cell lines resembled nutrient starvation-induced autophagy, displaying a noticeable enhancement. These findings suggest that the mutation of LHBs through N-linked glycosylation induces autophagy in hepatoma cells and may be involved in AMPK/mTOR signaling.

### N-linked glycosylation mutation of LHBs induces accumulation of autophagosomes

**Fig 4:**
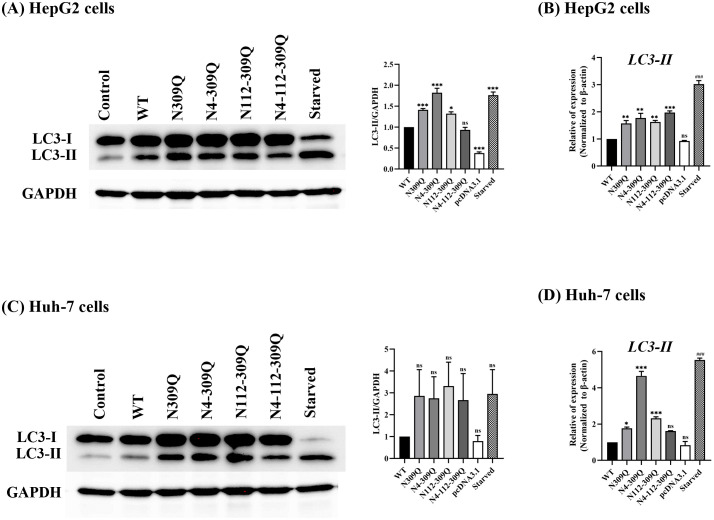
Effects of wild-type and LHBs mutants on LC3 conversion in transfected cells. (A) HepG2 cells and (C) Huh-7 cells were either starved or transfected with pcDNA3.1 plasmid (control) or a plasmid expressing the WT or LHBs mutants. Seventy-two hours post-transfection, total cell proteins were collected, and the expression levels of LC3-II were determined by western blotting. GAPDH was used as the protein loading control. The relative intensities of the bands were quantified by normalization to GAPDH using the ImageJ software. Levels of the corresponding mRNAs were determined by RT-qPCR using specific primers (B and D). Data are presented as the mean ± SEM of three independent experiments. *p < .05; **p < .01; ***p < .001; ^#^*p* < .05; ^##^*p* < .01; ^###^*p* < .001; ns, not significant.

To confirm that LHBs mutations at N309Q, N4-309Q, N112-309Q, and N4-112-309Q induce autophagosome formation, the conversion of LC3 from the cytosolic form (LC3-I) to the lipidated autophagosome-associated form (LC3-II) was analyzed by western blotting. Nutrient starvation, which induces autophagy, was included in the studies as a positive control. The mutated LHBs proteins at N309Q, N4-309Q, N112-309Q, and N4-112-309Q significantly increased the amount of LC3-II in human hepatoma HepG2 and Huh7 cells compared to WT LHBs-transfected cells, similar to the effect observed in starved cells (Fig 4A and 4C). Additionally, an increased amount of LC3-II was observed with nutrient starvation in both HepG2 and Huh7 cells compared to cells transfected with pcDNA3.1.

To provide further evidence of LHBs mutation-induced autophagosome formation, the mRNA expression level of LC3-II was detected using RT-qPCR. Compared with pcDNA3.1-transfected cells, an increase in the mRNA expression level of LC3-II was observed (Fig 4B and 4D).

Overall, these results suggest that the mutation of N-linked glycosylation in LHBs induces the accumulation of autophagosomes in hepatic cells.

### N-linked glycosylation mutation of LHBs impairs lysosomal acidification

**Fig 5:**
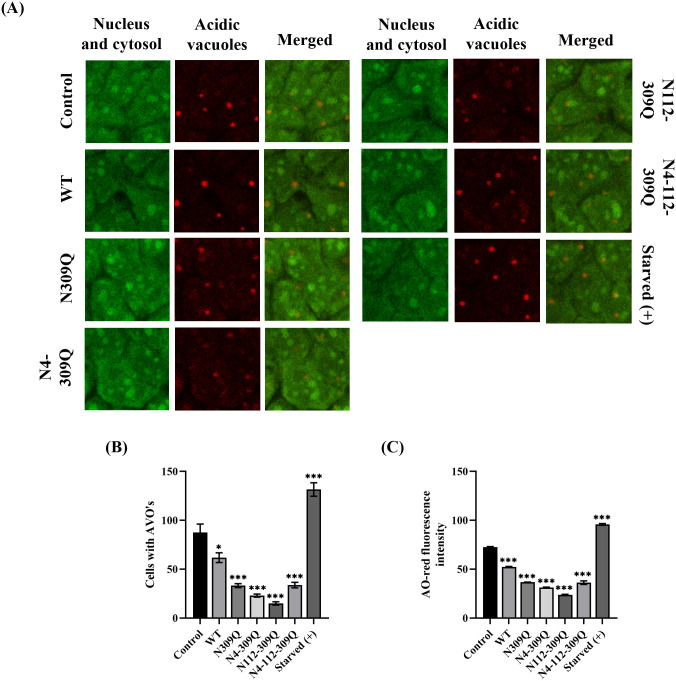
Detection and quantification of acidic vesicular organelles. (A) Immunofluorescence microscopy of acridine orange-stained Huh-7 cells starved for 6 hours with EBSS or transfected with pcDNA3.1 plasmid (control), WT, or LHBs mutants. An increase in the number of cells with AO accumulating acidic vesicular organelles (orange-red fluorescence) was evident. (B) Quantitation of cells with AVO’s from four to six random image fields, totaling approximately 150 cells in each image field. (C) Quantification of the fluorescence intensity in control and LHBs-transfected Huh-7 cells was performed using the ImageJ software. Values represent the mean ± SEM. *p < .05; **p < .01; ***p < .001.

Since acidification is required for the maturation and activation of lysosomal enzymes, the maintenance of acidity is a hallmark of functionality in mature lysosomes and autolysosomes. To further determine whether LHBs mutations at N309Q, N4-309Q, N112-309Q, and N4-112-309Q affect lysosomal acidification, the presence of acidic vacuolar organelles (AVO’s) in LHBs mutation-expressing cells was examined by assessing acridine orange (AO) retention. The non-protonated monomeric form of AO emits a green fluorescence in the cytosol and nucleus. However, when the dye enters acidic lysosomes, the protonated form is trapped in aggregates that fluoresce bright red (Fig 5A). LHBs mutations at N309Q, N4-309Q, N112-309Q, and N4-112-309Q decreased the number of cells with acidic vacuoles and red fluorescence intensity by 2.6-, 3.8-, 5.8-, and 2.5-fold and 2.0-, 2.3-, 3.0-, and 2.0-fold, respectively, compared to the control (Fig 5B and 5C), indicating a reduced number of acidified compartments. In contrast, starved Huh-7 cells exhibited 1.5- and 1.3-fold increases in the number of cells with acidic vacuoles and red fluorescence intensity, respectively, compared with the control.

Together, these results suggest that the N-linked glycosylation mutation in LHBs impairs lysosomal acidification in hepatic cells.

### N-linked glycosylation mutation of LHBs play a vital role in HBV replication, progeny secretion, and HBV gene expression

**Fig 6:**
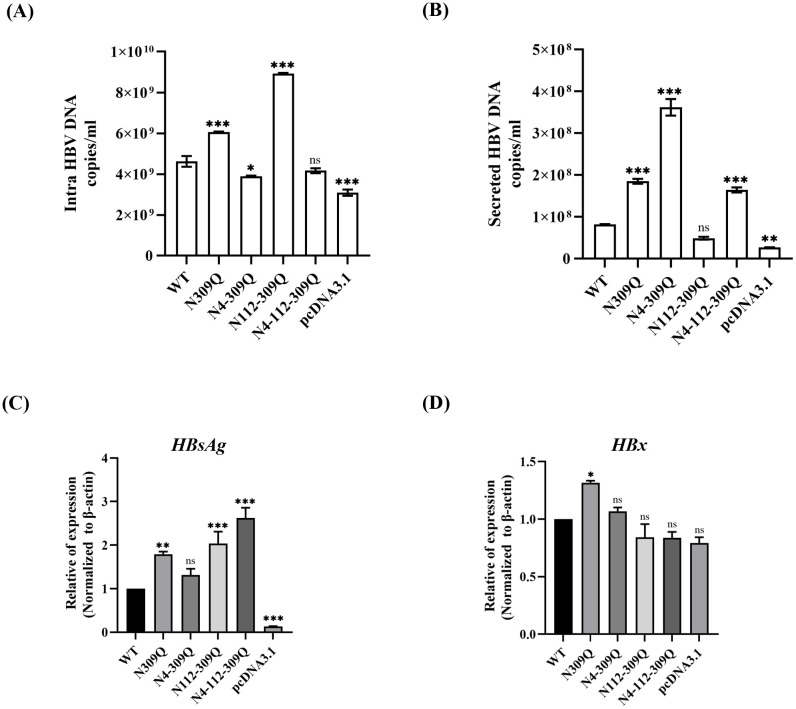
Impact of wild-type and LHBs mutants on HBV DNA replication, secretion, and gene expression. (A-B) HepG2.2.15 cells were transfected with plasmids expressing either WT LHBs or N-glycan mutants of LHBs and harvested after 72 hours. (A) Intracellular HBV DNA and (B) HBV progeny DNA from secreted particles were quantified using qPCR. (C-D) HepG2.2.15 cells were harvested 72 hours post-transfection for the detection of the relative expression levels of (C) HBsAg and (D) HBx using RT-qPCR. The data are plotted as mean and SEM. *p < .05; **p < .01; ***p < .001; ns, not significant.

Next, we investigated whether N-linked glycosylation mutation of LHBs regulates HBV replication in hepatoma cells. LHBs mutations at N309Q, N4-309Q, N112-309Q and N4-112-309Q were transfected into HepG2.2.15 cells with stable HBV replication. HBV replicative intermediates (HBV RIs) and secreted HBV DNA (in HBV virions) were isolated on day 3 after transfection and analyzed using qPCR. Compared to the control (pcDNA3.1), the amount of HBV RIs increased significantly after LHBs transfection. As compared to the WT, the N309Q mutant led to 1.3- and 2.3-fold increased levels of both intracellular and secreted HBV DNA, respectively, whereas the N4-309Q mutant significantly decreased HBV replication by 1.2-fold compared to the WT, but significantly promoted HBV secretion by 4.4-fold. In contrast, the N112-309Q mutant showed significantly enhanced intracellular HBV DNA levels (1.9-fold), but inhibited the secretion of HBV DNA. However, the N4-112-309Q mutant showed no significant difference in intracellular HBV DNA, but significantly increased the secretion of HBV DNA compared to the WT (2.0-fold) (Fig 6A and 6B). Furthermore, the N-linked glycosylation mutation of LHBs enhanced HBsAg mRNA expression in HepG2.2.15 cells (Fig 6C). We also observed that the LHBs mutant N309Q significantly increased HBx mRNA level in HepG2.2.15 cells (Fig 6D).

Together, these results suggest that N-linked glycosylation mutations effectively promote HBV replication, HBsAg and HBx gene expression, and progeny secretion, depending on the mutated N-linked glycosylation sites.

### N-linked glycosylation mutation of LHBs modulates HBV replication by regulating cellular autophagy

**Fig 7:**
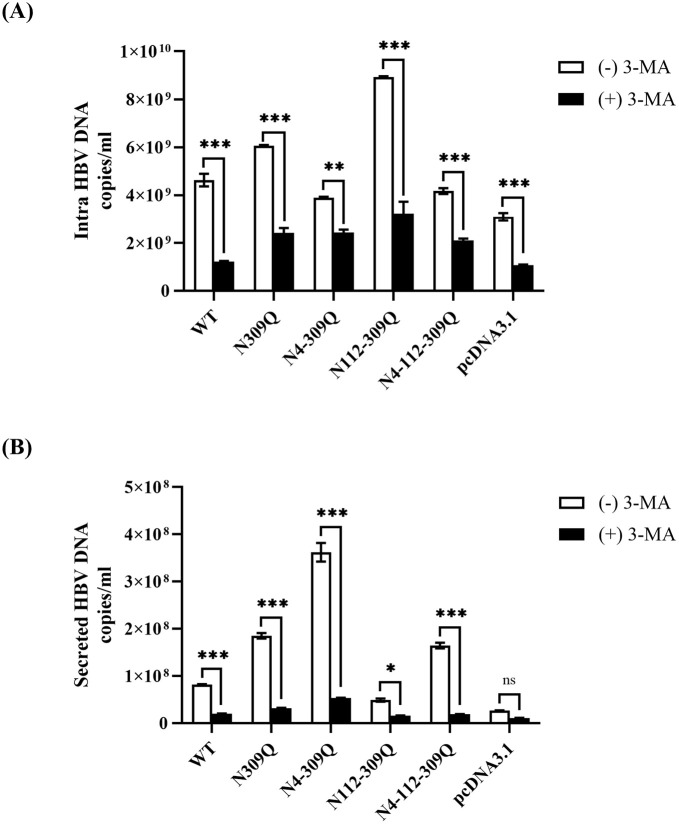
The effect of autophagy on HBV replication and secretion. (A-B) HepG2.2.15 cells were transfected with plasmids expressing either WT LHBs or N-glycan mutants of LHBs for 24 hours and further incubated with an autophagy inhibitor 3-MA for 48 hours. (A) These cells were then extracted HBV replicative intermediate from core particles and quantified by qPCR. (B) HBV progeny DNA in the supernatant was extracted and quantified by qPCR. The data are plotted as mean and SEM. *p < .05; **p < .01; ***p < .001.

Autophagy plays a positive role in HBV replication and assembly in hepatoma cells. In order to comprehend the beneficial impact of autophagy on HBV replication, HegG2.2.15 cells were transfected with plasmids that expressed WT LHBs or N-glycan mutants of LHBs for 24 hours. Subsequently, the cells were treated with 3-MA, an inhibitor of autophagy, for 48 hours, and the impact on HBV replication was assessed. Interestingly, when autophagy was inhibited by 3-MA, almost 50% of HBV DNA was still detectable (Fig 7A). This suggests that LHBs N-linked glycosylation mutations at positions N309Q, N4-309Q, N112-309Q, and N4-112-309Q may actively stimulate HBV replication and prompt cellular autophagy to work in concert with it.

qPCR analysis was also performed on secreted HBV progeny DNA to better understand the role of autophagy in HBV DNA. Similar to the intracellular HBV DNA levels, secreted HBV progeny DNA levels in the supernatant were dramatically reduced after 3-MA treatment (Fig 7B). These results imply that the induction of autophagy is required for the impact of LHBs N-linked glycosylation mutations on HBV secretion.

### N-linked glycosylation mutation of LHBs is unrelated to apoptosis

**Fig 8:**
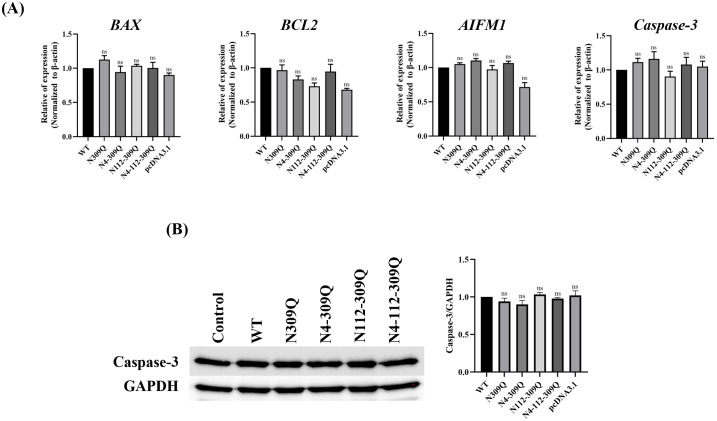
Apoptosis markers in HepG2.2.15 cells. (A) The relative mRNA levels of *BAX*, *BCL2*, *AIFM1* and *Caspase-3* were determined by RT-qPCR and (B) Immunoblot analysis of Caspase-3 in HepG2.2.15 cells were detected following 72 hours transfection. Data are plotted as the mean ± SEM. ns, not significant.

To detect whether the N-linked glycosylation mutation of LHBs influences apoptosis, we quantified the expression of apoptosis genes in HepG2.2.15 cells. The findings showed that after transfection with LHBs mutants in comparison to WT, the expression of the apoptosis gene remained unchanged (Fig 8A). Subsequently, the RT-qPCR results were confirmed with the protein levels of caspase-3, an executioner caspase that plays an important role in apoptosis and is a primary target for cancer treatment. No differences in the protein expression of caspase-3 were observed when comparing cells transfected with LHBs mutants to the wild-type (Fig 8B). In conclusion, these findings suggest that LHBs N-linked glycosylation mutations are not involved in triggering apoptosis.

## Discussion

N-linked glycosylation is a post-translational modification crucial for membrane protein folding, stability, and other cellular functions. Changes in glycosylation can modulate inflammatory responses, enable viral immune escape, and promote cancer cell metastasis [15]. Additionally, it has been reported that autophagy plays a prominent role in sustaining cell viability in cancer cells while averting apoptosis [31, 32].

HBV large surface protein consists of preS1, preS2, and S domains and plays pivotal roles in the morphogenesis of HBV and infection of hepatocytes. Recent studies have demonstrated that LHBs is modified by posttranslational N-linked glycosylation at asparagine 4, 112, and 309 of the preS1, preS2 and S domains, respectively [15]. In this study, we introduced single, double, and triple N-linked glycosylation mutations of LHBs at positions N4, N112, and N309 by amino acid substitution with glutamine. The LHBs protein was expressed in HEK293T cells and detected by western blotting. The results showed that LHBs proteins of the wild-type (WT) and the N4Q, N112Q, and N4-112Q mutants expressed two bands, which represent the glycosylated (gp42) and non-glycosylated bands (gp39), whereas the N309Q, N4-309Q, N112-309Q, and N4-112-309Q mutants expressed only a non-glycosylated band (gp39), suggesting that asparagine residue 309 is a crucial position in the N-linked glycosylation of LHBs. Furthermore, N-linked glycosylation of LHBs was confirmed by PNGase F treatment, an enzymatic process that removes N-linked glycans from glycoproteins. The observed differences in protein molecular weight following treatment with PNGase F confirmed that the detected shifts in molecular weight were indeed attributed to the N-linked glycosylation modification of the protein.

Earlier studies have indicated that HBV-mediated cell survival is linked to starvation-induced autophagy in hepatic cells. Both hepatitis B virus (HBV) and one of its encoded proteins, HBx and SHBs, have been shown to enhance the autophagic process to facilitate viral replication. However, the impact of N-linked glycosylation of HBV surface proteins on hepatocellular autophagy has not been explored. Our results demonstrate that N-linked glycosylation modification of LHBs is associated with hepatocellular autophagy, particularly at N-linked glycosylation site N309Q. Furthermore, combined mutations involving N309Q may contribute to this effect, potentially through the upregulation of the AMPK/mTOR signaling pathway.

Hepatitis B virus (HBV) has been shown to induce autophagosome accumulation, promoting viral DNA replication [30]. Several studies have demonstrated that autophagosome induction by HBV contributes to virus production, and inhibiting autophagosome formation can suppress HBV replication [25, 28, 33]. This suggests that these viruses have evolved mechanisms to exploit the autophagy machinery as proviral host factors that support viral replication. The increased number of autophagosomes is crucial for acquiring the viral envelope, indicating that membranes derived from autophagosomes may serve as a source of viral envelopment [30, 33]. In our study, we observed a significant increase in the levels of LC3-II in hepatoma cells transfected with LHBs mutants, including N309Q, N4-309Q, N112-309Q, and N4-112-309Q, suggesting enhanced autophagosome formation. Autophagic degradation can be inhibited by either suppressing lysosomal proteolytic activity (for example with chloroquine or glucosamine) or preventing autophagosome-lysosome fusion [34]. To further assess the impact of LHBs mutants on lysosomes, we examined lysosomal acidification in Huh-7 cells expressing LHBs mutants by evaluating the retention of acridine orange (AO). AO emits bright red fluorescence upon entering acidic lysosomes. Compared to starved cells or pcDNA3.1, we observed a dramatic decrease in cytoplasmic AO-red dots and AO-red intensity in LHBs-expressing Huh-7 cells. Additionally, a significant reduction in the number of AO-red dots and AO-red intensity was noted in the LHBs mutants compared to the WT, indicating impairment of lysosomal acidification by the LHBs mutants. This finding aligns with previous research demonstrating that HBV and HBx proteins disrupt lysosomal acidification without affecting autophagosome-lysosome fusion in Huh-7 cells, a condition favorable for HBV replication [1].

Glycosylation plays a crucial role in the biology of infection for many enveloped viruses, including hepatitis viruses. The glycosylation of transmembrane or surface glycoproteins is known to enhance correct folding, viral particle production, and release. However, alterations in glycosylation may impact interactions between ligands and receptors, influencing virus proliferation and infectivity [35]. Thus, N-linked glycosylation is essential for various biological functions of viruses. In this study, we investigated whether N-linked glycosylation mutations in LHBs affect HBV replication and secretion. Our observations revealed that LHBs mutants, especially N309Q and N112-309Q, significantly increased HBV DNA levels in HepG2.2.15 cells, while N4-309Q decreased HBV DNA compared to the WT. Interestingly, LHBs mutants, particularly N309Q, N4-309Q, and N4-112-309Q, significantly promoted HBV secretion, a process inhibited by N112-309Q. These findings suggest that N-linked glycosylation mutations in LHBs efficiently increase HBV replication and progeny secretion, depending on the location of mutant N-linked glycosylation.

HBV is a major risk factor for HCC development and is considered to trigger carcinogenesis through several molecular mechanisms [36]. Recent studies have shown that N-glycans on LHBs are associated with ER stress-mediated cell cycle dysregulation and cell proliferation, thereby triggering carcinogenic processes [22, 37]. Additionally, the expression of LHBs is maintained in HCC and likely plays an important role in promoting tumor progression. Immunohistochemical staining was performed to examine the expression of various surface proteins in surgically resected tumors from patients with HBV-related HCC. These data clearly demonstrated that LHBs is continuously expressed during carcinogenesis and probably plays an important role in cancer progression [38].

Of all the HBV genes, HBx has generated the most interest in HBV-related hepatocarcinogenesis. More than 95% of patients with HBV-related cirrhosis and dysplasia are positive for HBx, and 70% of patients with HBV-related HCC produce HBx [39]. Another in vitro study involving HepG2 hepatoma cells concluded that HBx-SMYD3 interaction, guided by the downstream target gene c-myc, promoted cell proliferation [40]. Additionally, HBx-transgenic mouse models show enhanced expression of fibroblast growth factor-inducible 14 (fn14) in c-myc/TGF-driven hepatocarcinogenesis [41]. HBx also disrupts cell cycle progression by upregulating p21 and p27 proteins that inhibit cyclin-dependent kinase (CDK) activity, which in turn enhances the ability of mitogen-activated protein kinase (MAPK) signaling to cause proliferation in hepatocytes [42]. In the present study, we found that N-linked glycosylation mutations in LHBs at N309Q, N112-309Q, and N4-112-309Q markedly increased HBsAg expression. Moreover, N309Q dramatically increased HBx gene expression in HepG2.2.15. These findings demonstrate that N-linked glycosylation mutations in LHBs, especially N309Q, might play a significant role in the regulation of HBV carcinogenesis.

While the close relationship between HBV replication and autophagy is acknowledged, the specific stages of the HBV life cycle influenced by autophagy remain incompletely understood. Several studies have suggested that autophagy induction contributes to virus production, and inhibiting autophagy significantly reduces HBV replication [34, 43, 44]. In this study, we aimed to elucidate the role of autophagy in regulating HBV replication in HepG2.2.15 cells transfected with LHBs mutants. To explore this, we concurrently treated the cells with the autophagy inhibitor 3-MA. Intriguingly, in HepG2.2.15 cells expressing LHBs mutants, intracellular HBV DNA was reduced and maintained at around fifty percent. Notably, treatment with 3-MA significantly decreased HBV DNA in the supernatant of HepG2.2.15 cells expressing LHBs mutants. These findings suggest that the influence of LHBs mutants on HBV replication and secretion hinges on the initiation of autophagy.

Apoptosis, programmed cell death, is a highly organized process serving as a protective strategy to maintain homeostasis in healthy organisms [45]. However, abnormal apoptosis can lead to immune escape, intracellular viral replication, and ultimately tumor formation [46]. Despite numerous studies aiming to elucidate the relationship between HBV infection and apoptosis, the results remain contradictory. In our present study, we observed that LHBs mutants did not exhibit any significant impact on apoptosis. HBV has been reported to promote the survival of HBV-infected hepatocytes under nutrient-deprived conditions by inhibiting apoptosis and activating autophagy. These studies suggest that HBx-induced autophagy is a cell-survival factor that facilitates persistent HBV infection [47]. It was previously shown that HBV replication and, in particular, the expression of the HBx protein transcribed from the viral genome during replication do not sensitize cells to apoptosis. Moreover, HBV needs to prevent apoptosis of its host hepatocytes to ensure the release of infectious progeny, thereby facilitating the virus spread within the liver [48].

In the present study, we demonstrated that single, double, and triple N-linked glycosylation mutations in LHBs, including N309Q, N4-309Q, N112-309Q, and N4-112-309Q, resulted in autophagosome accumulation and impaired lysosomal acidification, which may influence autophagic flux in hepatoma cells. Additionally, the LHBs mutants, N309Q and N112-309Q, enhanced HBV replication. Furthermore, N309Q, N4-309Q, and N4-112-309Q promoted the secretion of HBV progeny through their association with cellular autophagy. Moreover, N-linked glycosylation mutations in LHBs, particularly in N309Q, may be involved in the regulation of HBV carcinogenesis.

The findings of this study enhance our comprehension of the molecular mechanisms through which HBV maintains persistence in host cells. This research is pioneering in elucidating the impact of N-linked glycosylation mutations in LHBs on HBV-induced autophagy. Consequently, these glycans emerge as potential targets for the development of innovative treatment strategies for viral hepatitis. Moreover, we provide a novel clue for further investigation of hepatocellular carcinogenesis.

## Supporting information

S1 Fig Standard curve established for HBV DNA quantitation. (TIF)

S1 Data Quantitative observations of the figures. (XLSX)

S1 Raw images Original uncropped immunoblot images. (PDF)
