# Supplementary figures and images for: The N-linked glycosylation modifications in the hepatitis B surface protein impact cellular autophagy, HBV replication, and HBV secretion
Source: PLoS One. 2024 Mar 15;19(3):e0299403. doi: 10.1371/journal.pone.0299403 (PMC10942060; doi:10.1371/journal.pone.0299403)

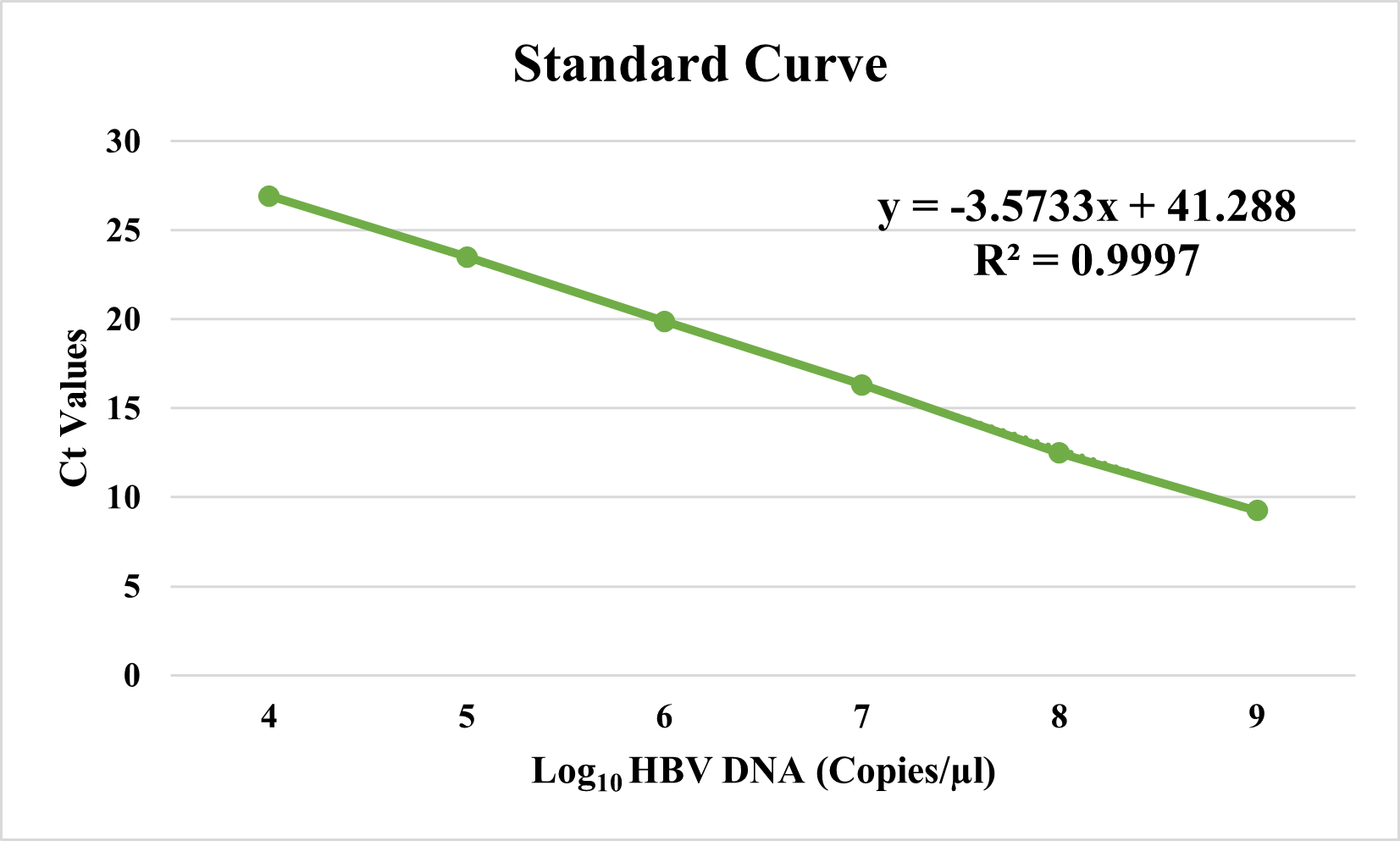

Supplement: S1 Fig — (TIF) [file pone.0299403.s001.tif]
