# Supplementary material for: The N-linked glycosylation modifications in the hepatitis B surface protein impact cellular autophagy, HBV replication, and HBV secretion
Source: PLoS One. 2024 Mar 15;19(3):e0299403. doi: 10.1371/journal.pone.0299403 (PMC10942060; doi:10.1371/journal.pone.0299403)

S1_Raw_images

Uncropped immunoblot images

Fig 1.

Lane 1: pcDNA3.1, Lane 2: wild-type, Lane 3: N4Q, Lane 4: N112Q, Lane 5: N309Q, Lane 6: N4-112Q, Lane 7: N4-309Q, Lane 8: N112-309Q, Lane 9: N4-112-309Q, Lane 10: protein ladder

LHBs protein before PNGase F incubation (gp42 kDa and p39 kDa)

Lane 1 2 3 4 5 6 7 8 9 10

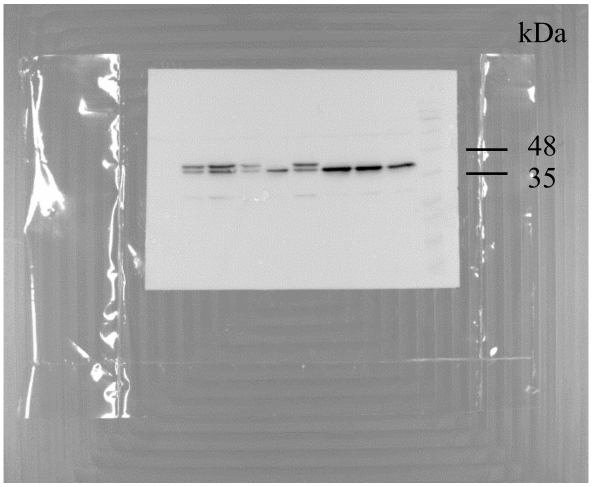

LHBs protein after PNGase F incubation (p39 kDa)

Lane 10 1 2 3 4 5 6 7 8 9

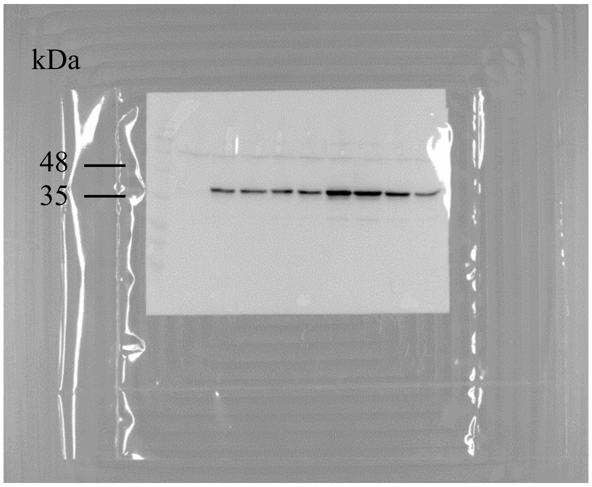

GAPDH loading control (37 kDa)

Lane 1 2 3 4 5 6 7 8 9 10

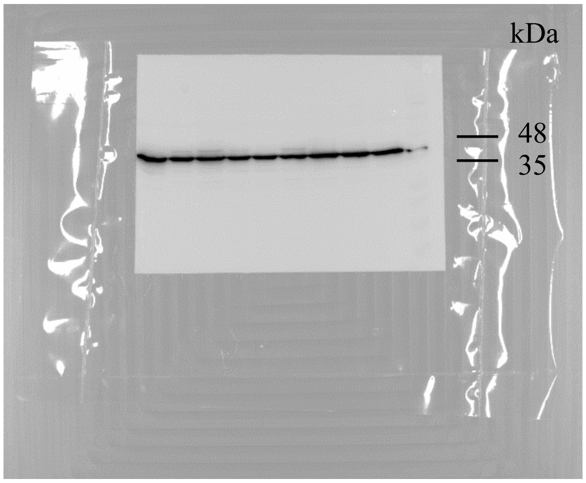

GAPDH loading control (37 kDa)

Lane 10 1 2 3 4 5 6 7 8 9

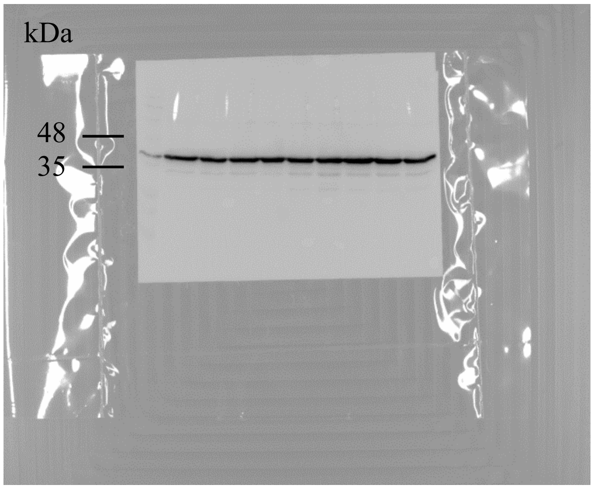

Fig 4A.

HepG2 cells

Repeat 1

Lane 1: protein ladder, Lane 2: pcDNA3.1, Lane 3: wild-type, Lane 4: N309Q, Lane 5: N4-309Q, Lane 6: N112-309Q, Lane 7: N4-112-309Q, Lane 8: starved cells

LC3 protein (LC3-I 16 kDa and LC3-II 14 kDa)

Lane 1 2 3 4 5 6 7 8

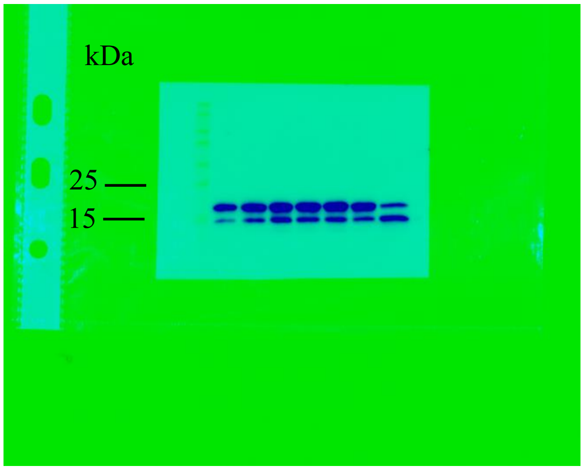

GAPDH loading control (37 kDa)

Lane 1 2 3 4 5 6 7 8

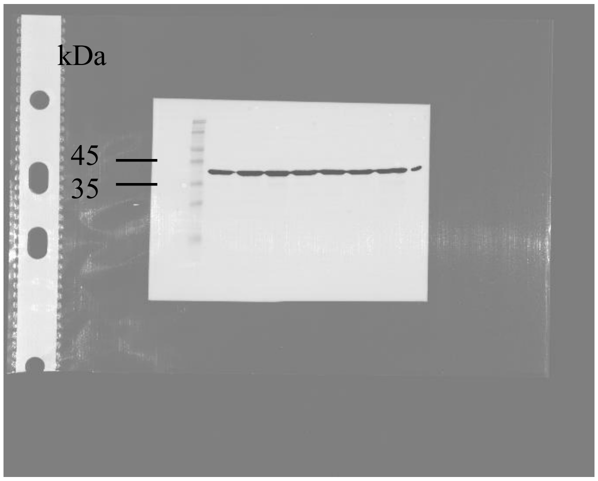

Lane 2 3 4 5 6 7 8

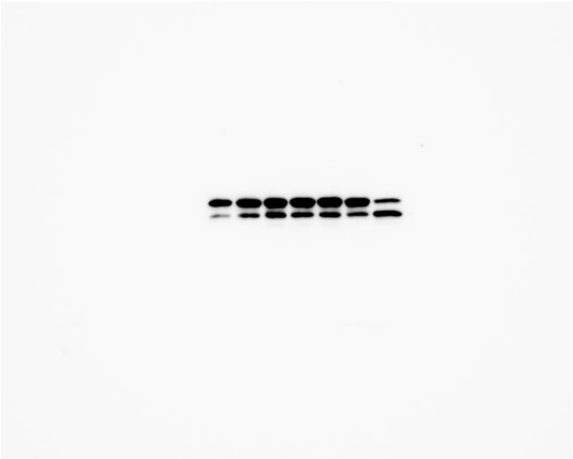

Lane 2 3 4 5 6 7 8

HepG2 cells

Repeat 2

Lane 1: protein ladder, Lane 2: pcDNA3.1, Lane 3: wild-type, Lane 4: N309Q, Lane 5: N4-309Q, Lane 6: N112-309Q, Lane 7: N4-112-309Q, Lane 8: starved cells

LC3 protein (LC3-I 16 kDa and LC3-II 14 kDa)

Lane 1 2 3 4 5 6 7 8

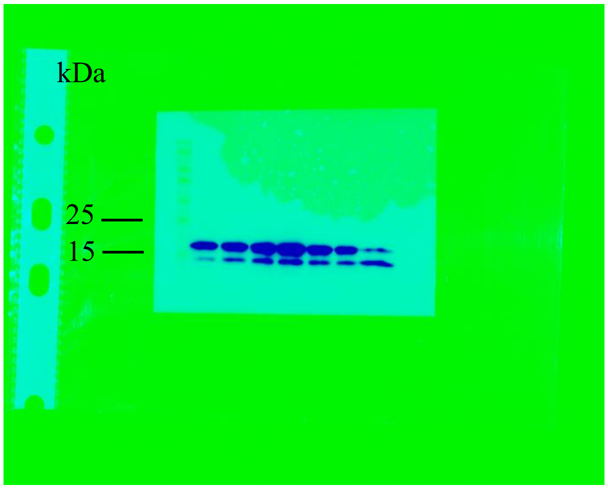

GAPDH loading control (37 kDa)

Lane 1 2 3 4 5 6 7 8

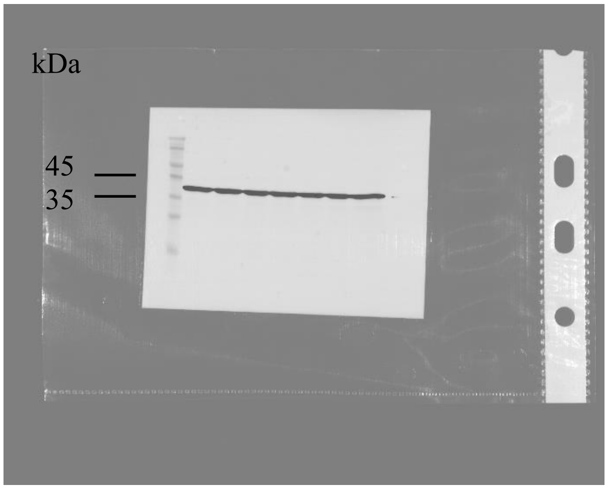

Lane 2 3 4 5 6 7 8

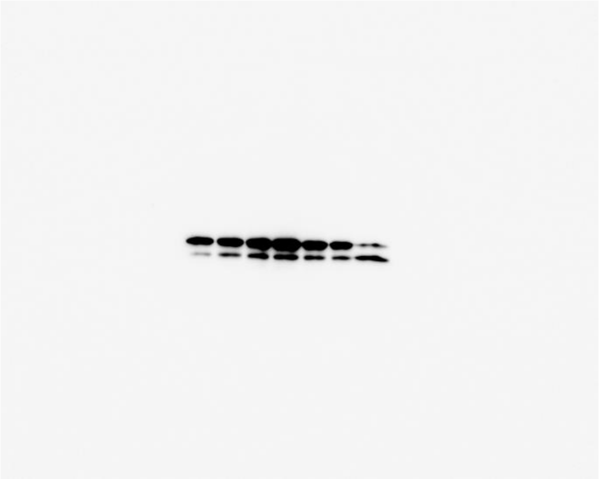

Lane 2 3 4 5 6 7 8

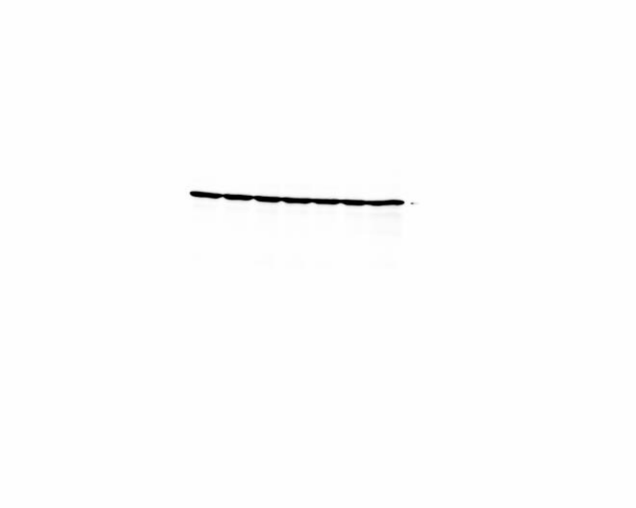

HepG2 cells

Repeat 3

Lane 1: protein ladder, Lane 2: wild-type, Lane 3: N309Q, Lane 4: N4-309Q, Lane 5: N112-309Q, Lane 6: N4-112-309Q, Lane 7: pcDNA3.1, Lane 8: starved cells

LC3 protein (LC3-I 16 kDa and LC3-II 14 kDa)

Lane 1 2 3 4 5 6 7 8

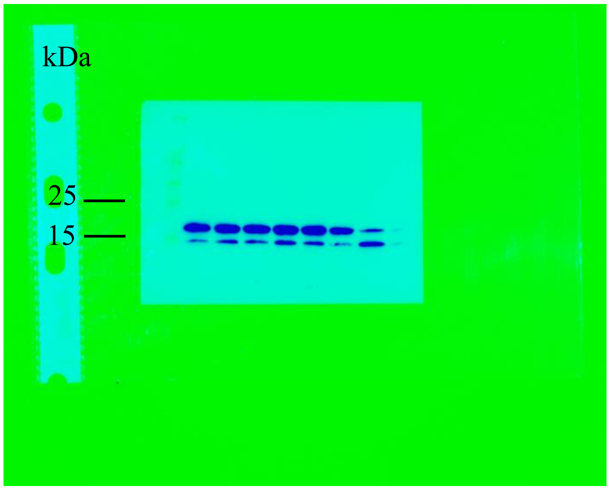

GAPDH loading control (37 kDa)

Lane 1 2 3 4 5 6 7 8

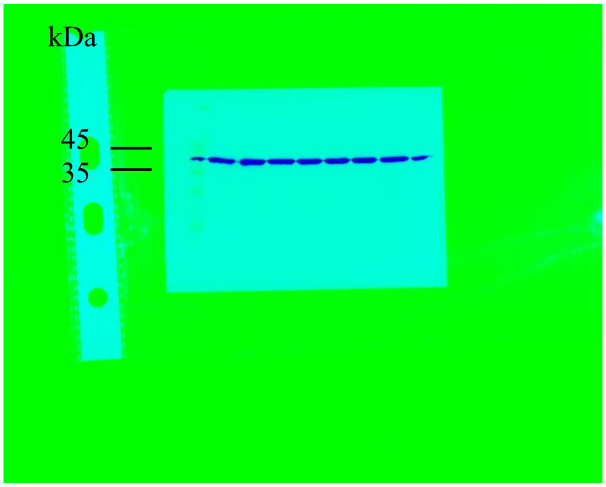

Lane 2 3 4 5 6 7 8

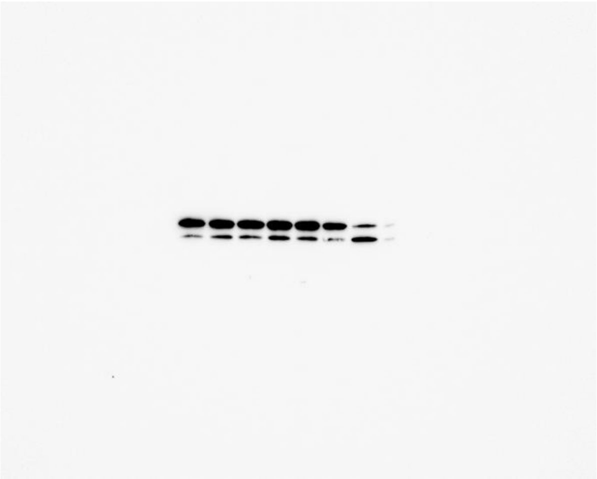

Lane 2 3 4 5 6 7 8

Fig 4C.

Huh-7 cells

Repeat 1

Lane 1: protein ladder, Lane 2: pcDNA3.1, Lane 3: wild-type, Lane 4: N309Q, Lane 5: N4-309Q, Lane 6: N112-309Q, Lane 7: N4-112-309Q, Lane 8: starved cells

LC3 protein (LC3-I 16 kDa and LC3-II 14 kDa)

Lane 1 2 3 4 5 6 7 8

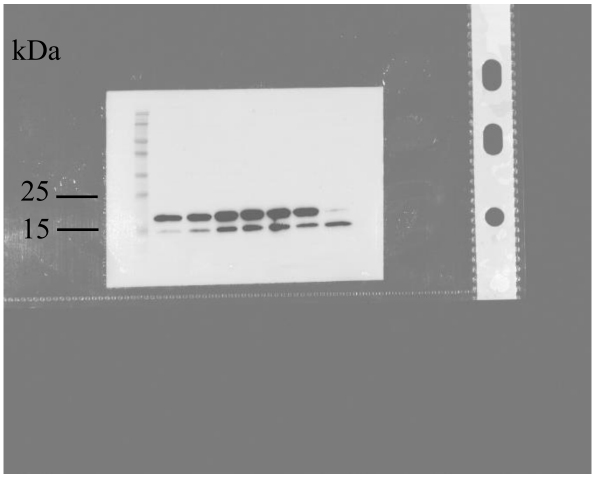

GAPDH loading control (37 kDa)

Lane 1 2 3 4 5 6 7 8

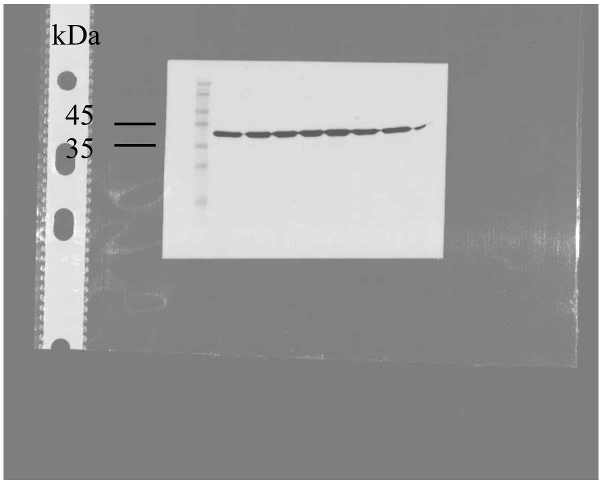

Lane 2 3 4 5 6 7 8

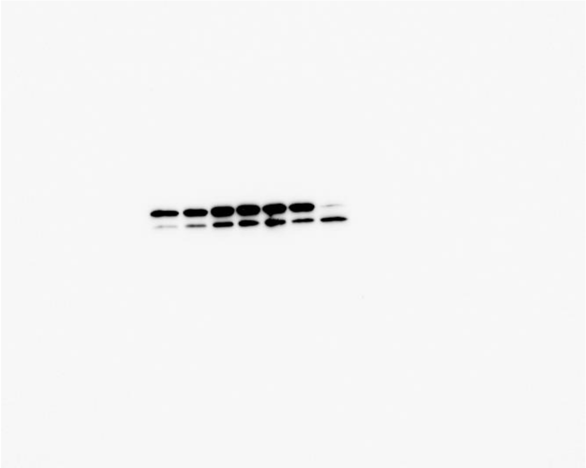

Lane 2 3 4 5 6 7 8

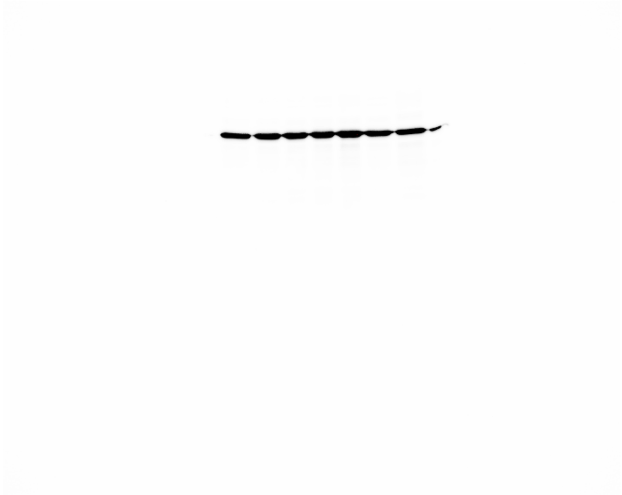

Huh-7 cells

Repeat 2

Lane 1: protein ladder, Lane 2: pcDNA3.1, Lane 3: wild-type, Lane 4: N309Q, Lane 5: N4-309Q, Lane 6: N112-309Q, Lane 7: N4-112-309Q, Lane 8: starved cells

LC3 protein (LC3-I 16 kDa and LC3-II 14 kDa)

Lane 1 2 3 4 5 6 7 8

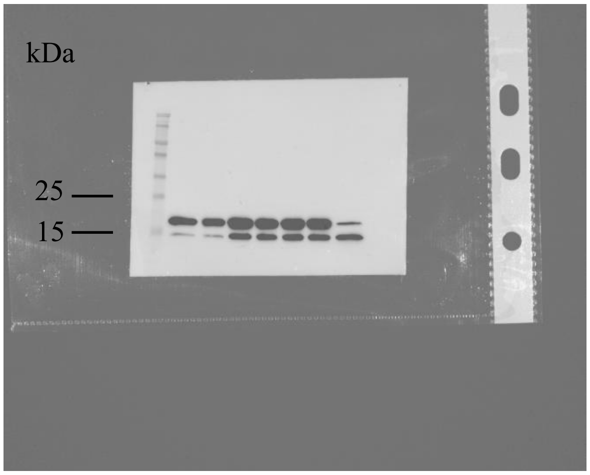

Lane 2 3 4 5 6 7 8

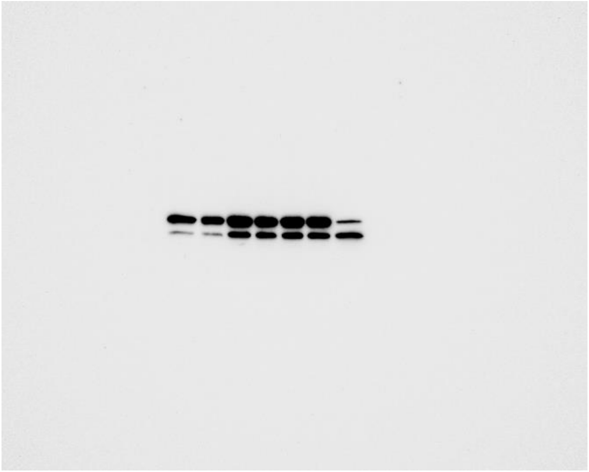

GAPDH loading control (37 kDa)

Lane 1 2 3 4 5 6 7 8

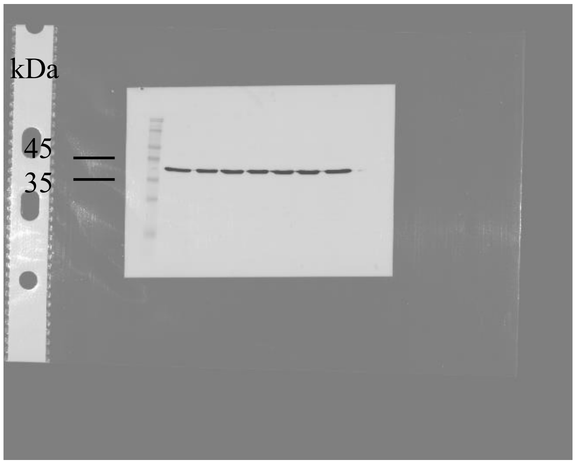

Lane 2 3 4 5 6 7 8

Huh-7 cells

Repeat 3

Lane 1: protein ladder, Lane 2: pcDNA3.1, Lane 3: wild-type, Lane 4: N309Q, Lane 5: N4-309Q, Lane 6: N112-309Q, Lane 7: N4-112-309Q, Lane 8: starved cells

LC3 protein (LC3-I 16 kDa and LC3-II 14 kDa)

Lane 1 2 3 4 5 6 7 8

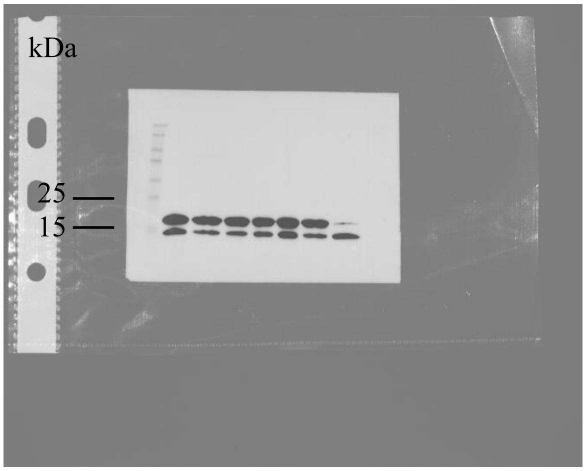

Lane 2 3 4 5 6 7 8

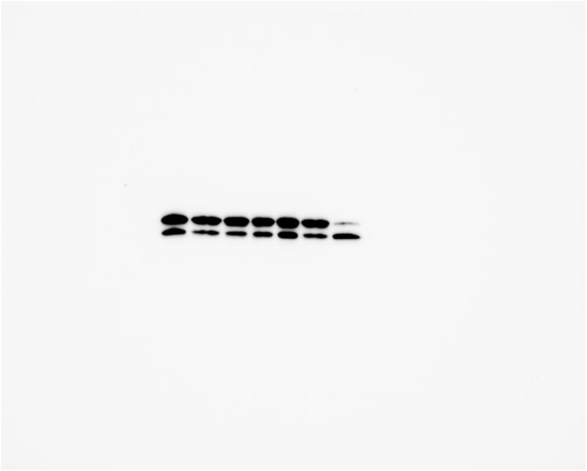

GAPDH loading control (37 kDa)

Lane 1 2 3 4 5 6 7 8

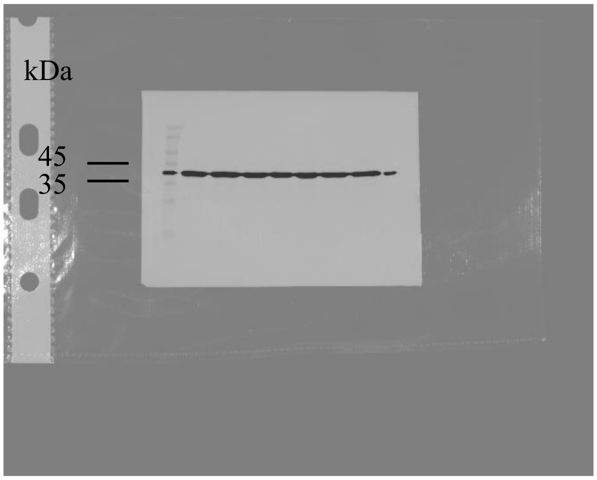

Lane 2 3 4 5 6 7 8

Fig 8B.

HepG2.2.15 cells

Repeat 1

Lane 1: protein ladder, Lane 2: pcDNA3.1, Lane 3: wild-type, Lane 4: N309Q, Lane 5: N4-309Q, Lane 6: N112-309Q, Lane 7: N4-112-309Q

Caspase-3 protein (35 kDa)

Lane 1 2 3 4 5 6 7

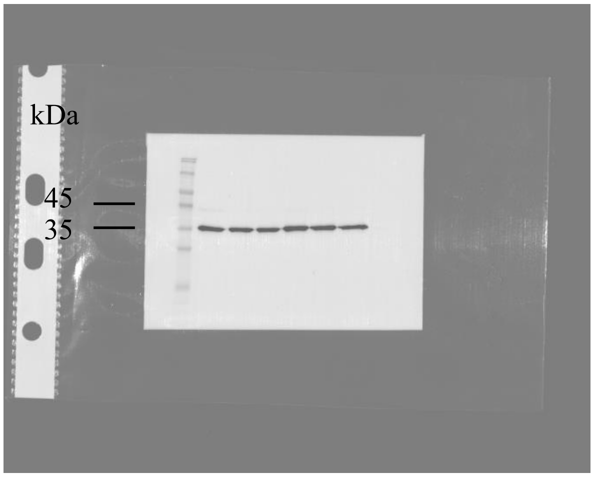

GAPDH loading control (37 kDa)

Lane 1 2 3 4 5 6 7

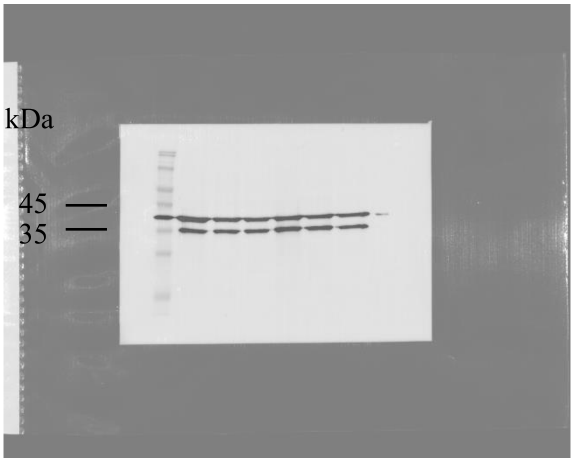

Lane 2 3 4 5 6 7

Lane 2 3 4 5 6 7

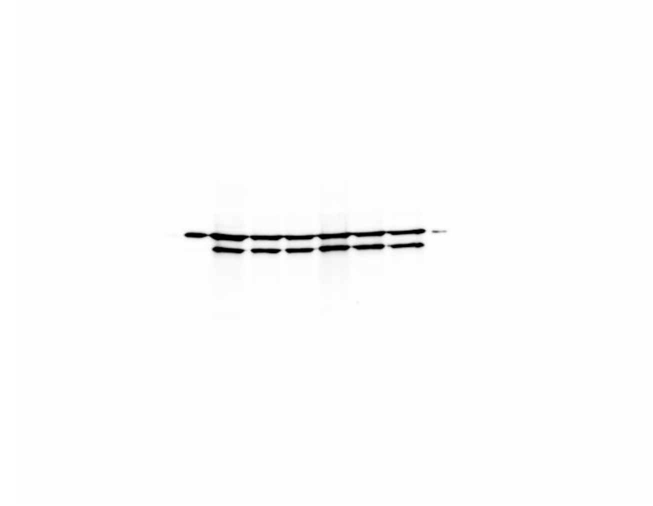

HepG2.2.15 cells

Repeat 2

Lane 1: protein ladder, Lane 2: pcDNA3.1, Lane 3: wild-type, Lane 4: N309Q, Lane 5: N4-309Q, Lane 6: N112-309Q, Lane 7: N4-112-309Q

Caspase-3 protein (35 kDa)

Lane 1 2 3 4 5 6 7

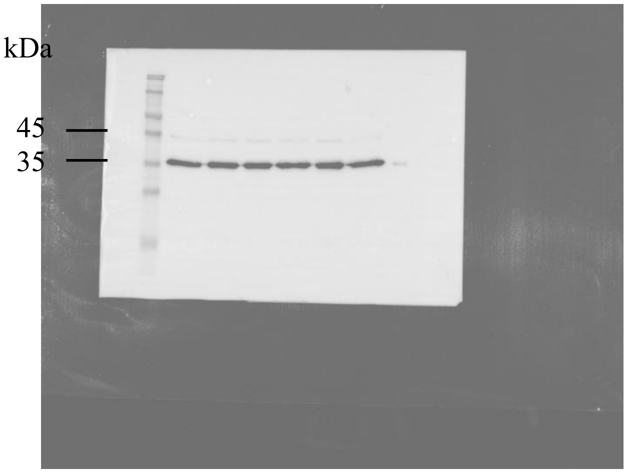

GAPDH loading control (37 kDa)

Lane 1 2 3 4 5 6 7

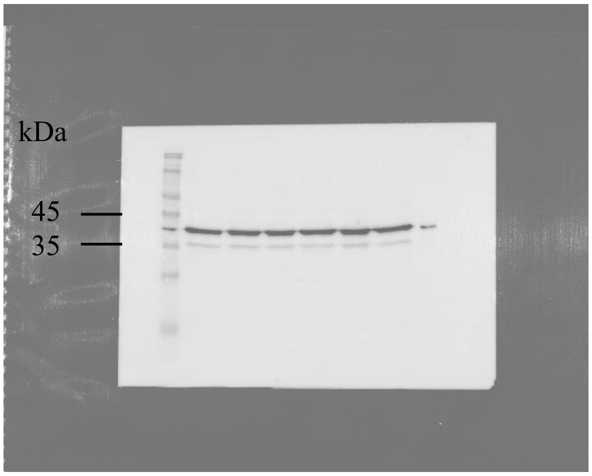

Lane 2 3 4 5 6 7

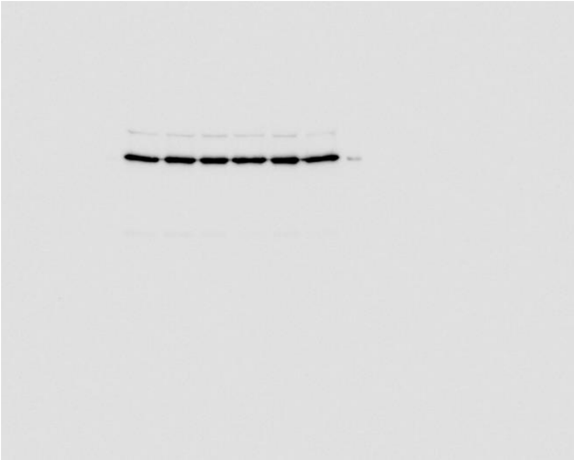

Lane 2 3 4 5 6 7

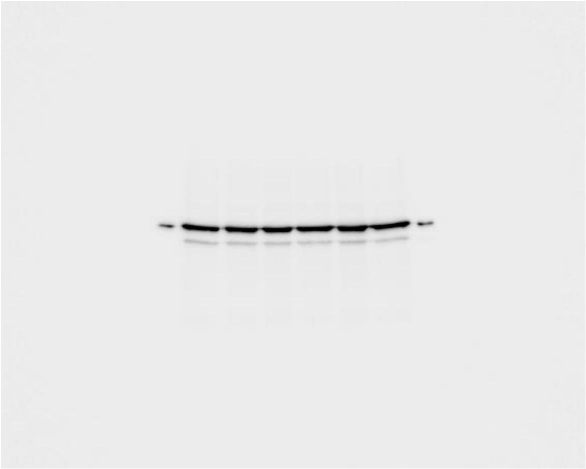

HepG2.2.15 cells

Repeat 3

Lane 1: protein ladder, Lane 2: pcDNA3.1, Lane 3: wild-type, Lane 4: N309Q, Lane 5: N4-309Q, Lane 6: N112-309Q, Lane 7: N4-112-309Q

Caspase-3 protein (35 kDa)

Lane 1 2 3 4 5 6 7

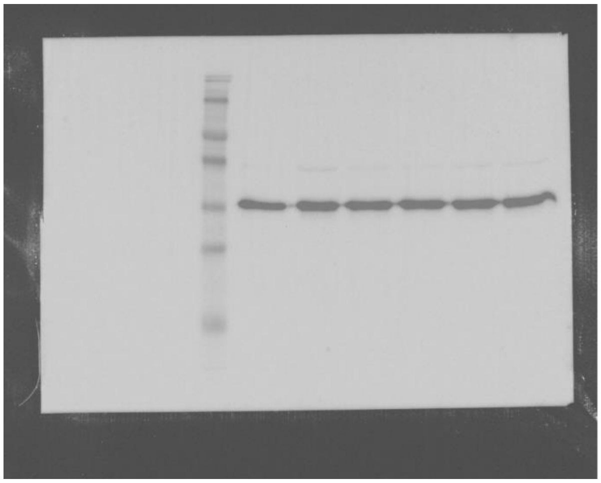

GAPDH loading control (37 kDa)

Lane 1 2 3 4 5 6 7

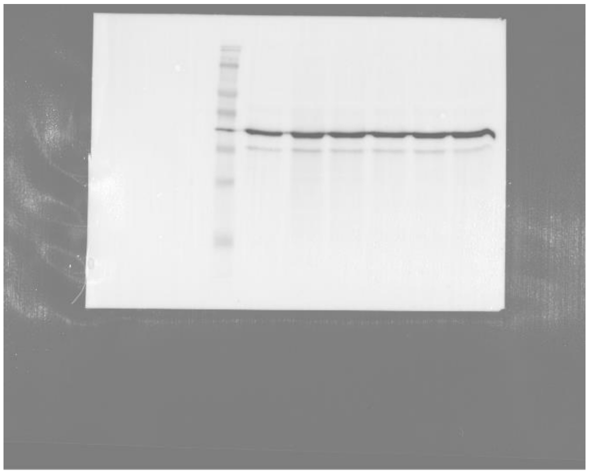

Lane 2 3 4 5 6 7

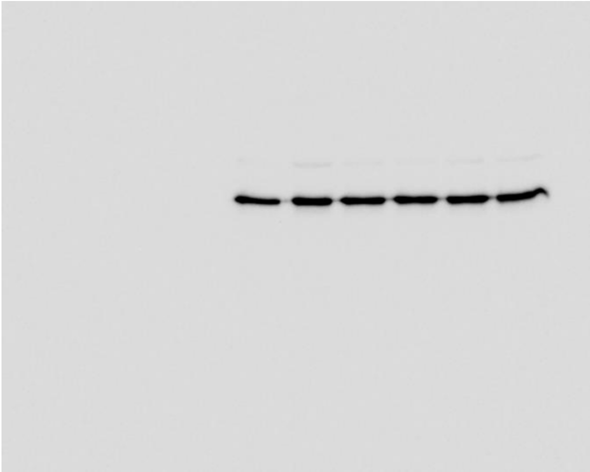

Lane 2 3 4 5 6 7

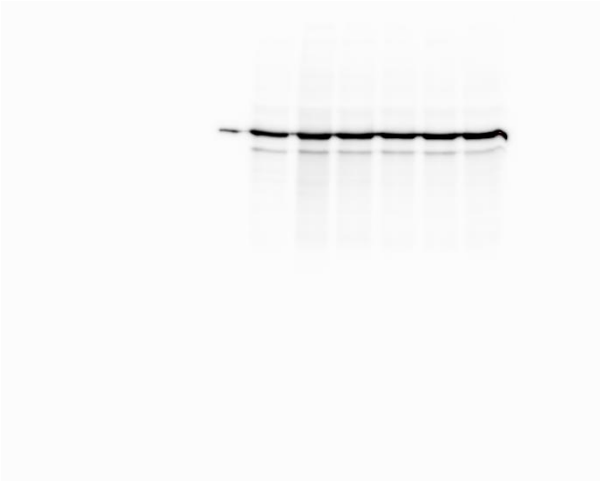

Supplement: S1 Raw images — (PDF) [file pone.0299403.s003.pdf]
